# Do telemonitoring projects of heart failure fit the Chronic Care Model?

**DOI:** 10.5334/ijic.1178

**Published:** 2014-07-17

**Authors:** Evi Willemse, Jef Adriaenssens, Tinne Dilles, Roy Remmen

**Affiliations:** Department of Health and Wellbeing, Thomas More University College, Turnhout, Belgium; Faculty of Medicine and Health Sciences, University of Antwerp, Belgium; Department of Health and Wellbeing, Thomas More University College, Turnhout, Belgium; Department of Health and Wellbeing, Thomas More University College, Lier, Belgium; Faculty of Medicine and Health Sciences, Centre for Research and Innovation in Care (CRIC), University of Antwerp, Belgium; Department of Primary and Interdisciplinary Care, Faculty of Medicine and Health Sciences, Centre for General Practice, University of Antwerp, Belgium

**Keywords:** primary health care, nursing evaluation research, chronic care model, telemedicine, heart failure, patient-centred care

## Abstract

This study describes the characteristics of extramural and transmural telemonitoring projects on chronic heart failure in Belgium. It describes to what extent these telemonitoring projects coincide with the Chronic Care Model of Wagner.

**Background:**

The Chronic Care Model describes essential components for high-quality health care. Telemonitoring can be used to optimise home care for chronic heart failure. It provides a potential prospective to change the current care organisation.

**Methods:**

This qualitative study describes seven non-invasive home-care telemonitoring projects in patients with heart failure in Belgium. A qualitative design, including interviews and literature review, was used to describe the correspondence of these home-care telemonitoring projects with the dimensions of the Chronic Care Model.

**Results:**

The projects were situated in primary and secondary health care. Their primary goal was to reduce the number of readmissions for chronic heart failure. None of these projects succeeded in a final implementation of telemonitoring in home care after the pilot phase. Not all the projects were initiated to accomplish all of the dimensions of the Chronic Care Model. A central role for the patient was sparse.

**Conclusion:**

Limited financial resources hampered continuation after the pilot phase. Cooperation and coordination in telemonitoring appears to be major barriers but are, within primary care as well as between the lines of care, important links in follow-up. This discrepancy can be prohibitive for deployment of good chronic care. Chronic Care Model is recommended as basis for future.

## Introduction

### 

#### Background

The ageing of the population and an increase in chronic diseases are challenges for the future [1–4]. In a typical European country, the share of 80 plus increases from 4% in 2005 to 6% in 2020 [5]. In 2025, this will rise further to 6.5% [6]. In 2060, the share of 65 plus will be 26%, of which 10% would have reached the age of 80 years and more [7]. Multi-morbidity will increase and will use a bigger share of the resources in the health system [5, 8]. Chronic heart failure is one of these chronic diseases with high morbidity, high mortality and high health-care costs [9]. It is characterised by periods of symptom worsening. It is estimated that 15% of the people over 80 will develop heart failure [10]. Optimal use of lifestyle measures, pharmaceuticals and monitoring is necessary [10–12].

A shift from secondary care to primary care is therefore imposing. Many health systems focus on the increasing share of care in the community, rather than treating people in more expensive intramural care [2, 13, 14]. Besides economic motives, quality improvement in home care has shown to result in a better outcome such as a decrease in the number of readmissions and an increase in the quality of life [15–17].

As a result of this shift, there is a need for new organisational structures and new partnerships [9]. The Chronic Care Model of Wagner describes the necessary components for quality of care. This model summarises the basic elements for improving care in health systems at the community, organisation, practice and patient levels. The dimensions of the Chronic Care Model are: the community, the health system, self-management support, delivery system design, decision support and clinical information systems [4].

The purpose of this model is to optimise the chronic care management. There are many deficiencies in the current management of chronic care, i.e. lack of coordination and patients are inadequately trained to manage their illnesses. Overcoming these deficiencies will require nothing less than a transformation of health care, from a system that is essentially reactive to one that is proactive and focused on keeping a person as healthy as possible [4].

When medical care wants to focus on social and societal aspects, the coordination of care and multidisciplinary collaboration is indispensable. In this model, a proactive approach with self-management and supporting information technologies has a prominent place.

A multidisciplinary approach of self-care and self-management, monitoring of clinical parameters and the process of the disease and the accessibility of health-care professionals may have beneficial effects on the outcome of patients with chronic heart failure [1, 9, 18–20]. Telemonitoring can hereby be used as a tool.

#### Context

The health systems of the European countries are becoming more complex and care expenditures are increasing [7, 21]. In the case of Belgium, the share of health care in GDP (10.2%) is among the highest in the EU countries (8.3%) and is still increasing [22]. The Belgian health care is regulated by federal and regional legislation. All Belgians are obliged to join a central care insurance. The focus in the current health-care organisation is located in the intramural sector. As a result of an increase in chronic care, a shift of health care has become necessary: cure has to make place for care [2, 21, 23]. Belgian medical practitioners have great therapeutic freedom. The free choice of the patient and the fee-for-service payment are central to the health-care system [7]. Patients in Belgium participate in health-care financing through official co-payments and various supplements [7]. The Flemish and the Central Government play an important economic role in the organisation of this system.

A large share of primary care physicians work in solo practice [7]. Only a small proportion of General Practitioners are grouped into multidisciplinary practices. Nursing home care is organised by home-care organisations, which have their origin in political compartmentalisation on the one hand, and by self-employed nurses on the other hand.

The role and organisation of the professional in the first and second lines of health care should be reviewed [9]. It is important that health professionals spend most of their time on tasks that require their expertise. This calls for a shift of some tasks from more specialised professionals to less-qualified professionals, resulting in lowering of health-care cost [9]. Collaboration between general practitioners and hospitals needs further research [24, 25]. The use of telemonitoring within these new cooperation structures is in line with the policy objectives of the government [26].

#### Telemonitoring as an innovative tool

Telemedicine being a subset of telehealth, uses communications networks for delivery of health-care services and medical education from one geographical location to another [27]. Telemedicine refers to the delivery of medical health services at a distance; there is no single or uniform telemedicine application [27]. Telemonitoring, within the concept of telemedicine is the (continuous or intermittent) observation of specific parameters of a patient from a physical distance. Clinical parameters which are measurable are stored, interpreted and reported. Telemonitoring can be organised not only in hospitals but also at home. Parameters (among others) can be heart rate, blood pressure, weight, specific items from electrocardiography, oxygen saturation and electro encephalography. Data can be transferred by telephone or by a wireless connection from the patient to a monitor [2, 3].

Telemonitoring can contribute to a new approach of developing integrated care. It offers an innovative and supportive technology that can be an added value for the reorientation of the care process [10, 11, 28]. Telemonitoring is one of the strategies to follow the treatment of chronic heart failure at home [11]. Telemonitoring may offer many advantages. Numerous national and international studies describe a significant improvement in outcomes of chronic heart failure, such as a decrease in the number of readmissions and an increase in quality of life [10, 11, 29–33]. Telemonitoring can reduce mortality and hospitalisations in patients with chronic heart failure and may increase the quality of life and patient satisfaction [11]. A systematic review describes a 34% reduction in risk of all-cause mortality and a 9% reduction of all cause hospitalisation with telemonitoring comparing usual care [11]. Chronic heart failure-related hospitalisation was reduced by 21% with telemonitoring comparing usual care [11].

#### Telemonitoring supporting self-management

Home monitoring extends from promotion of self-care and home visitations to telemedicine [1]. Self-care is the decision-making process patients use to maintain physiological stability. Self-care includes multiple components, such as adhering to medications, following diet and exercise recommendations, and actively monitoring for congestion. Self-management is therefore a complex process: patients have to recognise a change in themselves (e.g., increasing oedema), evaluate the symptom, decide to take action, implement a treatment strategy (e.g., taking an extra diuretic dose) and evaluate the response to therapy [1]. Self-care and self-management behaviours are ultimately the responsibilities of the patient, even if they are frequently seen in the office or telephoned at home. A multidisciplinary approach to support self-care and self-management in patients with chronic heart failure is important [1]. A randomised control trial described the effects of tailored telemonitoring on heart failure patients’ knowledge, self-care, self-efficacy and adherence [34]. Tailored telemonitoring was found to educate patients with heart failure and to improve their self-care abilities and sense of self-efficacy. [34]. Telemonitoring is an upcoming research topic and a promising tool to support self-management of chronic care. Autonomy and promotion of self-care for the patient and his/her informal caregiver are important in chronic care [9].

#### Telemonitoring in Belgium

Given the concerns about ageing, increasing chronic illness and the shortage in the number of nurses and physicians in various developed countries, alternative ways to establish efficient interactions between patients and caregivers are explored [9, 34, 35]. In Belgium, the Federal Health-care insurance (National Institute for Health and Disability Insurance) called for projects in the program “Prioritise chronic illness”. Funding for projects was provided. Also regional governments supported telemonitoring projects. Furthermore, some projects have been funded by industrial partners.

#### Telemonitoring and Chronic Care Model

Quality of life and high-quality care for patients suffering from chronic diseases as heart failure are characterised by productive interactions between caregivers and patients that consistently provide the assessments support for self-management, optimisation of therapy, and follow-up associated with good clinical and patient outcomes [36]. Telemonitoring is seen as a viable new approach to managing chronic illness. However, strategies in deploying new technologies might have some deficiencies. New technology means more than the use of tools [37, 38]. Telemonitoring research projects particularly focus on improved outcomes (hospital (re-)admissions and length of hospital stay) [36]. Attention is required for the capabilities to support patients with chronic illness better. The Chronic Care Model of Wagner describes system changes associated with improvements in chronic illness care [38, 39]. Into a framework, by use of dimensions, quality improvement of chronic care is guided [4].

## The study

### Aim

The aim of the study was to describe the extramural projects in telemonitoring of chronic heart failure in Belgium (Flanders). We checked if the elements of the Chronic Care Model had been taking into account and how the implementation processes proceeded. This study also discusses the role of the clinical coordination of the projects and takes an in-depth look at the multidisciplinary approach.

### Design

We studied the prevalence of telemonitoring in heart failure through the systematic research of available sources (document analysis). First, scientific publications were searched. Second, search engines (Google, Google scholar) and grey literature (presentations from symposia, …) were used to find all telemonitoring projects in Belgium (Flanders). Third, key informants were approached by contacting heart failure clinics and home-care organisations. At last, a snowball method was used to find other key informants.

Subsequently, we contacted key informants and examined the process-based approach of the projects by using a structured telephone and face-to-face interview of project managers of each project. Eleven key informants were contacted of which seven were nurses (three heart failure nurse, two nurses at home and two project leading nurses in home care), one project member (not a physician or nurse but other education), one physician and two project leaders (nursing education but not working as a nurse in the field).

The interview had a duration of maximum one hour. The preference went to a face-to-face interview. If transportations and appointment (face to face) were not possible, a telephone interview was conducted.

First, projects were situated into their context. Attention was paid to the project descriptions and their objectives, funding sources and job descriptions of the participating partners. Second, the different phases of the projects were described by the key informant. Third, experiences from out of the pilot projects were shared. Thereafter, project information was compared with the dimensions of the Chronic Care Model: (1) resources and policies, (2) self-management support, (3) organisations of health care, (4) delivery system design, (5) decision support and (6) clinical information systems. Structured questions with regard to the dimensions were made. Peer debriefing occurred between members of the research team while analysing project information.

Afterwards, project leaders were subsequently contacted again with the aim to check the specific project information and link this with the domains of the Chronic Care Model. The collected data were, in a final phase for each project, thus resubmitted to the project managers for the final adjustments (member checking).

The data collection was carried out between 2 January 2012 and 31 October 2012. Data were analysed in categories, according to the project content and the dimensions of the Chronic Care Model.

### Results

Seven projects were linked with the domains of the Chronic Care Model. These projects met the criteria of telemonitoring projects with non-invasive monitoring in patients with chronic heart failure.

Table 1 gives an overview of the main features.

Table 1 describes the characteristics of the telemonitoring projects in Belgium (Flanders). Seven projects were described.

The projects had a duration of three months up to three years. The questions and the intended outcome of the projects were mainly clinically oriented.

To understand the aims of the project, we looked at the difference of the projects. The objectives of the projects were varied. Projects from secondary health care (C, E and F) aimed, by experimental trials (randomised control trial), to describe the effectiveness of telemonitoring. Project E studied the effect of the use of telemonitoring. In a second project (F), not only outcome measures, such as mortality and hospitalisations, were followed but also the way in which the alarms were generated. A first step towards cooperation between primary and secondary care was provided (F).

Primary care projects described the feasibility (B), the value of technology (A) and the way in which communication between the different care providers should take place (B).

Aspects that were examined included the identification of predictors of heart failure (C), a decrease in the deterioration of heart failure (D, G), a reduction of the causes of hospitalisation and the number of readmissions (D, F and G), and a decrease in mortality (E, F and G). In two projects, where the feasibility of telemonitoring at home was examined, the focus was also on cooperation and communication (A and B).

The coordination of four of the projects was managed from out of secondary care (C, D, E and F). Two of the completed projects were managed from out of primary care (A and B). Both projects involved cooperation with General Practitioners in primary care, as well as support by a specialist in secondary health care.

Financing of the projects was provided by federal bodies (B, C, D, E and F), regional bodies (A and G), industrial partners (C) or private resources (A and B).

Meanwhile, three projects have been completed (A, B and E). The two projects from primary care (A and B) were still in the evaluation phase. The results of the survey on patient satisfaction and quality of life, which had yet to be processed, would help to determine the start of a new project or extension of the existing project. Project E took place in secondary health care. Invasive telemonitoring was coordinated and monitored. A non-invasive telemonitoring study was conducted using allocated research funding. Scientific publications of the study results were submitted and published for two projects only [29, 40].

### Description of the projects on the basis of the Chronic Care Model

Table 2 describes the seven telemonitoring projects on the basis of the dimensions of the chronic care model.

#### Community

##### Resources and policies

A rethinking of the approach towards chronic care is organised on the one hand through pilot projects and on the other hand by sensitisation and information.

Provision of funding for the start-up of multiple projects and the use of Internet and digital communication makes it possible to facilitate the research of telemonitoring in heart failure. To execute the projects, funding was provided by the province (A), National Institute for Health and Disability Insurance (Social Security; B, D, E and F), industrial partners (C) and public funding (C and G). Project A also used its own resources for the development of the pilot project. Two projects were entirely embedded in primary care (A and B). Patients in the pilot phase were sensitised and informed about the possibilities of telemonitoring for the monitoring of their chronic disease.

##### Self-management support

Projects A and B focused on self-management. However, in practice, it was difficult to implement this in routine care. A short project time made it also difficult to measure the increase in the patient's understanding of his/her disease and the presence of increased self-management.

The focus in the projects was primarily on monitoring and recording parameters. Telephone support and the ability to contact a monitored chronic heart failure patient were used to support self-management directly or indirectly.

Project A visualised self-management by use of questionnaires. This approach was used to find out how the self-understanding had changed in patients through regular confrontation with the measured values. Project B focused on patient satisfaction. Projects from the second line (C, E and F) made a standard protocol available for the patient.

#### Health Systems

##### Organisation of health care

By starting up telemonitoring projects, organisations show a willingness to deal with the care for the chronic patient. Unfortunately, transmural care was not fully optimised. The contacts with the managers revealed that coordination primarily took place within the responsible institution. The cooperation between the different lines of care was complex. The involvement of the General Practitioners in these projects remained low. Bottlenecks with respect to digital communication, cooperation and coordination were found. The follow-up of patients occurred within the own institution. Transmural data sharing was difficult and did not happen between the care boundaries.

At the start-up of the projects, goals per project were determined by defining patient inclusion criteria. A precise medical screening of the target population was performed in each of the projects.

Coordination of projects A and B took place in primary care. The coordination and responsibility of these projects occurred by primary care organisations. The objective to be achieved was not only a clinical outcome but the optimisation of transmural communication between health-care professionals. Project B emphasised the follow-up of chronic monitoring rather than acute monitoring.

In Projects C, E and F, coordinated in secondary health care, a clinical outcome was set out as a target. Project C tried to generate an algorithm for the follow-up of alarm parameters.

Coordination in Projects E and F took place in the first as well in the second line. The focus was solely on the follow-up of alarm parameters.

##### Delivery system design

Follow-up of the monitoring was done by a standard procedure for every pilot project. This standard procedure described the project content and project progress. The standard procedures, along with the accessibility of health-care professionals, made it possible to deliver efficient and effective care in a proactive manner. Care professionals involved in all projects were trained in the use of monitoring. In none of the projects, new job or task content of nursing was created for the development of the project. In both the projects from primary care (A and B) and in the projects from secondary health care (C, E and F), the new care was added as a supplement to routine care.

Project A selected home-care nurses for the follow-up of telemonitoring. Project B recruited reference nurses in geriatrics. In both projects, the nurse was the central contact person for the patient. The nurse was responsible for the installation of the equipment, the monitoring of parameters and the contacts with the head of the institution and the General Practitioners.

The General Practitioner was involved in these projects in order to select patients and to titrate medication.

In Projects E and F, the heart failure nurse was the central contact person for the patient. This nurse was always available for formal and informal information provision related to illness, telemonitoring and follow-up of the clinical status of the patient. This nurse cooperated with the cardiologist. Project C collaborated with an external firm for the technical support of the equipment at home. The nurse in this project recorded only the generated alarms. In the projects from secondary health care, no additional support at home was given. The General Practitioner was minimally involved in these projects.

##### Decision support

The majority of projects were based on the existing guidelines on the treatment and follow-up of patients with heart failure and / or hypertension [12]. A link to evidence-based practice would be a major advantage but was not provided in all projects. Only in two projects (A and B), a flow chart was developed for the follow-up of parameters and generated alarms. One project had the aim to develop an algorithm (C).

No mandatory therapy protocol was outlined from the studies that were coordinated from primary care (A and B). An individualised care plan was developed, based on the specific objectives that the chronically ill patient wanted to achieve by means of a conversation with the General Practitioner and the home nurse. The informal caregivers were also involved if possible, for example, in determining the general condition of the patient and his habits.

In the projects from the second line (C, E and F), the patient had the possibility to contact the doctor and/or heart failure nurse in the hospital ward. Information on the use of the equipment and guidelines for the monitoring of the parameters was given at discharge from the hospital.

##### Clinical information systems

Bottlenecks related to digital communication caused difficulties in both intra-and extramurally cooperation. Data storage occurred, for the devices from different industrial partners, on a separate platform. For these different industrial partners, the health-care professional needed to login with a different account. The use of technology that allows recording and sending patient data also included the safe storage of these individual patient data. Also, registered data could not be integrated in the patient record or the medical file.

In all projects, support was offered by companies specialised in telemonitoring technology. In five of these started projects, the equipment was provided for free during the project (A, C, E and F). In Project B, equipment was purchased for the project. These devices were given to the General Practitioners for further use after completion of the project. The projects from secondary health care also used other equipment for the follow-up of invasive telemonitoring. Nevertheless, none of the projects managed to integrate the recorded parameters in the electronic patient record. Each company offered its own platform to distribute the generated data. Yet, Projects A and B tried to share information between the care boundaries. Communication from home-care organisations and General Practitioners to the hospital was present.

### Discussion

This study describes seven projects of telemonitoring in patients with chronic heart failure in Belgium (Flanders) on the basis of the Chronic Care Model. Table 3 gives an overview of the barriers and success factors for the implementation of telemonitoring projects.

Two projects were described in a report or a scientific publication [29, 40]. Two projects were still in the start-up phase (in November 2012). Different partners were involved in all these projects. Funding was mainly provided by the National Institute for Health and Disability Insurance (Social Security). Additional funding was received from industrial companies, financed by research budgets or from own resources. The duration of the projects was highly dependent on the number of enrolled patients. The project time was extended when the sample size was too low.

Projects were often not integrated into the existing health-care field. The care that was offered within telemonitoring did not take into account the provided routine care. This implied that the projects had no correlation with the standard care received by a chronic patient. The generated data were not a part of the records of the home-care nurses and General Practitioners. Data were presented on a separate platform. All these factors were an important obstacle for the integration of this innovative form of care in existing health-care systems. Projects had therefore rather a mono-dimensional design.

Within the projects that started-up in primary care, attempts were made to involve the patient by the joint production of an individualised care plan. These projects tried to involve family and informal caregivers. However, the extent to what telemonitoring met the individual needs of the patient and family was never mentioned. Future projects should therefore also take into account the following questions: Who needs to be monitored? Are informal caregivers involved and informed?

Anticipation to the individual health-care needs, by providing tailored care, and by increasing the understanding of chronic illness care of the elderly patient and informal caregivers, is an important key element of the Chronic Care Model. The need for acute or chronic monitoring should therefore be viewed critically. The focus of telemonitoring should not be solely on the parameters, but must take into account all the aspects of the chronic patient. Difficulties, in managing the patient as a whole, occur in the current health-care system where there is therapeutic freedom and fee for service.

Care professionals were involved in only three of the five projects from primary care while the provision of telemonitoring took place in the home situation. Coordination in secondary health care was done in chronic heart failure centres by heart failure nurse. Only in one project, the General Practitioner was actively involved. In the two primary care projects, there was a close cooperation between the home nursing services and general practitioners in the region. Independent home nursing and home-care nurses from other organisations were a rarely involved party. All these factors were counter-productive for multidisciplinary and transmural cooperation.

The cooperation between the hospital and the General Practitioners was not optimal. A major reason quoted by General Practitioners and specialists from secondary health care was the lack of funding. Telemonitoring is an “additional task” and comes on top of the daily care for the patient. The success of the project also depends heavily on the motivation and commitment of both health-care professional and the patient. The start-up of monitoring, the monitoring itself and the recording were additional tasks for the doctor and nurse who were not reimbursed at the time of the project. Therefore, they experienced it as an extra time-consuming task. The funding structure plays an important role in the success or failure of such initiatives. The current financing of the Belgian health-care providers is based on a “pay per act”. For telemedicine and telemonitoring, there is currently no financial structure and there is even no conclusive legal framework, which can lead to liability problems. Follow-up studies or projects of telemonitoring in chronic heart failure can therefore only be executed when there is proper financial support and when proper incentives are provided for professional groups that are involved in the projects. Nevertheless, this study provides information on barriers and problems in setting up these telemonitoring projects.

#### Limitations

This research has attempted to describe all the relevant chronic heart failure telemonitoring projects in Belgium (Flanders). All of these projects were small scale and were embedded in their specific contexts. The generalisability of the results of the projects in our study is therefore rather limited. The reported data are based on the perception of the health-care professionals. The opinions of individual professionals, the chronic patient and their informal carers have not been addressed.

Nevertheless useful lessons for the Belgian systems can be learned from out of these projects.

## Conclusion

Not all the projects initiated to accomplish all of the dimensions of the Chronic Care Model. Cooperation between the first and second lines was still not optimal. The monitoring of parameters instead of the monitoring of the chronic patient was emphasised, and the chronic patient was inadequately involved in the process.

Four out of the seven projects were initiated from hospitals and had mainly a clinical focus; the three projects in primary care focused especially on the communication between health-care professionals. The involvement of the informal carers was highly variable and appeared to be better developed in the studies with a strong primary focus. None of the projects had continuity after the pilot phase.

A key success factor for these telemonitoring initiatives is the integration within a broader change framework. The Chronic Care Model can provide this framework and has a significant added value in the design of projects in the future and in supporting inter-professional care.

## Figures and Tables

**Table 1.**
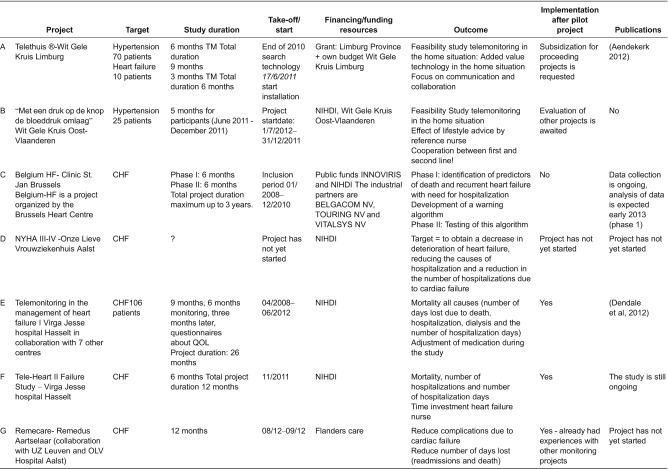
Characteristics of the chronic heart failure projects in Flanders

Abbreviations: TM = Telemonitoring, CHF = Chronic Heart Failure, NIHDI = National Institute for Health and Disability Insurance.

**Table 2.**
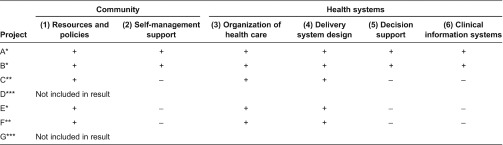
Description of the seven telemonitoring projects on the basis of the dimensions of the Chronic Care Model.

*Studies already completed, **Study finds itself now in phase I, ***Study is not yet launched/started.

(1) resources and policies: +: Receiving funding (see Table 1) and sensitisation and information/−: No funding.

(2) self-management support: +: Telephone support and contact moments and questionnaires/−: only one or two of previous items are present.

(3) organisation of health care: +: Intention to move care from hospital to home/−: no home care present-patient is followed in the hospital.

(4) delivery system design: +: Standard procedures with project content and project process and training −: no procedures.

(5) decision support: +: Flow charts based on existing guidelines with transmural communication −: no transmural communication within the use of flow charts.

(6) clinical information system: +: Access of digital communication between boundaries −: access of digital communication only in own organisation.

**Table 3.**
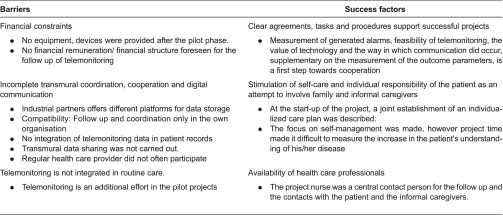
Overview of the barriers and success factors of the telemonitoring projects in Belgium (Flanders).
